# Correction: Benchmarking of survival outcomes following haematopoietic stem cell transplantation: A review of existing processes and the introduction of an international system from the European Society for Blood and Marrow Transplantation (EBMT) and the Joint Accreditation Committee of ISCT and EBMT (JACIE)

**DOI:** 10.1038/s41409-019-0740-9

**Published:** 2019-11-21

**Authors:** John A. Snowden, Riccardo Saccardi, Kim Orchard, Per Ljungman, Rafael F. Duarte, Myriam Labopin, Eoin McGrath, Nigel Brook, Carmen Ruiz de Elvira, Debra Gordon, Hélène A. Poirel, Francis Ayuk, Yves Beguin, Francesca Bonifazi, Alois Gratwohl, Noel Milpied, John Moore, Jakob Passweg, J. Douglas Rizzo, Stephen R. Spellman, Jorge Sierra, Carlos Solano, Fermin Sanchez-Guijo, Nina Worel, Andreu Gusi, Gillian Adams, Theodor Balan, Helen Baldomero, Gilles Macq, Evelyne Marry, Florence Mesnil, Elena Oldani, Rachel Pearce, Julia Perry, Nicole Raus, Urs Schanz, Steven Tran, Leonie Wilcox, Grzegorz W. Basak, Christian Chabannon, Selim Corbacioglu, Harry Dolstra, Jürgen Kuball, Mohamad Mohty, Arjan Lankester, Sylvia Montoto, Arnon Nagler, Jan Styczynski, Ibrahim Yakoub-Agha, Regis Peffault de Latour, Nicolaus Kroeger, Ronald Brand, Liesbeth C. de Wreede, Erik van Zwet, Hein Putter

**Affiliations:** 1 Department of Haematology, Sheffield Teaching Hospitals NHS Foundation Trust, Sheffield, UK; 2 Haematology Department, Careggi University Hospital, Florence, Italy; 3 Department of Haematology, Southampton General Hospital, Southampton, UK; 4 Department of Cellular Therapy and Allogeneic Stem Cell Transplantation, Karolinska University Hospital, Division of Hematology, Department of Medicine Huddinge, Karolinska Institutet, Stockholm, Sweden; 5 Servicio de Hematologia y Hemoterapia, Hospital Universitario Puerta de Hierro Majadahonda, Madrid, Spain; 6 EBMT Study Office, Hôpital Saint Antoine, Paris, France; 7 EBMT Executive Office, Barcelona, Spain; 8 EBMT Registry Office, London, UK; 9 Belgian Cancer Registry, Brussels, Belgium; 10 Department of Stem Cell Transplantation, University Medical Center Hamburg, Hamburg, Germany; 11 Department of Haematology, CHU and University of Liège, Liège, Belgium; 12 Institute of Hematology “Seràgnoli”, S. Orsola-Malpighi University Hospital, Bologna, Italy; 13 Department of Hematology, Medical Faculty, University of Basel, Basel, Switzerland; 14 CHU Bordeaux, service d’hematologie et therapie Cellulaire, F-33000 Bordeaux, France; 15 Department of Haematology, St Vincent’s Hospital Sydney, Darlinghurst, NSW Australia; 16 EBMT Activity Survey office and Swiss National Transplant Registry SBST, Basel University Hospital, Switzerland, Basel Switzerland; 17 Center for International Blood and Marrow Transplant Research, Milwaukee, WI USA; 18 Center for International Blood and Marrow Transplant Research, Minneapolis, MN USA; 19 Hematology Department, Hospital Sant Pau, Barcelona, Spain; 20 Servicio de Hematología, Hospital Clínico de Valencia, Valencia, Spain; 21 IBSAL, Hospital Universitario de Salamanca, Salamanca, Spain; 22 Blood Group Serology and Transfusion Medicine, Medical University of Vienna, Vienna, Austria; 23 Department of Biomedical Data Sciences, Leiden University Medical Center, Leiden, The Netherlands; 24 Agence de la biomédecine, Paris, France; 25 Italian National BMT Registry (GITMO), Bergamo, Italy; 26 BSBMT Data Registry, London, UK; 27 Société Francophone de Greffe de Moelle et de Thérapie Cellulaire (SFGM-TC) Centre Hospitalier Lyon Sud, Pierre-Bénite, France; 28 Haematology, University Hospital, Zurich, Switzerland; 29 Australasian Bone Marrow Transplant Recipient Registry (ABMTRR), Darlinghurst, NSW Australia; 30 Department of Hematology, Oncology and Internal Medicine, Medical University of Warsaw, Warsaw, Poland; 31 Institut Paoli Calmettes & Centre d’Investigations Cliniques en Biothérapies, Marseille, France; 32 Department of Pediatric Hematology, Oncology and Stem Cell Transplantation, University of Regensburg, Regensburg, Germany; 33 Department of Laboratory Medicine, Radboud University-Nijmegen Medical Centre, Nijmegen, The Netherlands; 34 Department of Haematology, University Medical Center Utrecht, Utrecht, The Netherlands; 35 Service d’Hematologie Clinique, Saint-Antoine Hospital, AP-HP, Sorbonne University, and INSERM UMRs 938, Paris, France; 36 BMT Centre, Leiden University Hospital, Leiden, The Netherlands; 37 St. Bartholomew’s and The Royal London NHS Trust, London, UK; 38 Chaim Sheva Medical Center, Tel-Hashomer, Israel; 39 Collegium Medicum, Nicolaus Copernicus University, Bydgoszcz, Poland; 40 CHU de Lille, Université de Lille, Lille, France; 41 Saint Louis Hospital, Paris Diderot University, Paris, France

**Keywords:** Haematological cancer, Haematopoietic stem cells

**Correction to: Bone Marrow Transplantation**

10.1038/s41409-019-0718-7

Following publication, it was noted that some author names were misspelt. Jan Styczinski has been corrected to Jan Styczynski, Regis Peffault de la Tour has been corrected to Regis Peffault de Latour and Gzregorz Basak has been corrected to Grzegorz W. Basak. This has been updated in the HTML and PDF versions of this article.

